# The Association of Mismatch Repair Status with Microscopically Positive (R1) Margins in Stage III Colorectal Cancer: A Retrospective Cohort Study

**DOI:** 10.1245/s10434-024-15595-0

**Published:** 2024-06-21

**Authors:** Henry G. Smith, Nis H. Schlesinger, Deepthi Chiranth, Camilla Qvortrup

**Affiliations:** 1 Abdominalcenter K, Copenhagen University Hospital – Bispebjerg and Frederiksberg, Copenhagen, Denmark; 2 Department of Pathology, Copenhagen University Hospital – Rigshospital, Copenhagen, Denmark; 3 Department of Oncology, Copenhagen University Hospital – Rigshospital, Copenhagen, Denmark

**Keywords:** Colon cancer, Rectum cancer, Mismatch repair status, Microscopically positive resection, Surgery

## Abstract

**Background:**

There is mounting evidence that microscopically positive (R1) margins in patients with colorectal cancer (CRC) may represent a surrogate for aggressive cancer biology rather than technical failure during surgery. However, whether detectable biological differences exist between CRC with R0 and R1 margins is unknown. We sought to investigate whether mismatch repair (MMR) status differs between Stage III CRC with R0 or R1 margins.

**Methods:**

Patients treated for Stage III CRC from January 1, 2016 to December 31, 2019 were identified by using the Danish Colorectal Cancer Group database. Patients were stratified according to MMR status (proficient [pMMR] vs. deficient [dMMR]) and margin status. Outcomes of interest included the R1 rate according to MMR and overall survival.

**Results:**

A total of 3636 patients were included, of whom 473 (13.0%) had dMMR colorectal cancers. Patients with dMMR cancers were more likely to be elderly, female, and have right-sided cancers. R1 margins were significantly more common in patients with dMMR cancers (20.5% vs. 15.2%, *p* < 0.001), with the greatest difference seen in the rate of R1 margins related to the primary tumour (8.9% vs. 4.7%) rather than to lymph node metastases (11.6% vs. 10.5%). This association was seen in both right- and left-sided cancers. On multivariable analyses, R1 margins, but not MMR status, were associated with poorer survival, alongside age, pN stage, perineural invasion, and extramural venous invasion.

**Conclusions:**

In patients with Stage III CRC, dMMR status is associated with increased risks of R1 margins following potentially curative surgery, supporting the use of neoadjuvant immunotherapy in this patient group.

## Introduction

Microscopically positive (R1) margins are defined as the presence of viable cancer cells ≤1 mm of the surgical margin and may be related to the primary tumor (R1tumor) or lymph node metastases/tumour deposits (R1LNM).^1^ Regardless of their subtype, R1 margins are associated with poorer oncological outcomes in patients with either colon or rectum cancers.^2–8^ Despite their recognized importance, there is an ongoing debate about whether R1 margins represent a technical failure at the time of surgery or are in fact a surrogate for more aggressive disease.^9–11^

Recent studies from our group based on a large cohort of patients with Stage III colorectal cancer have provided several lines of evidence to support the latter argument. In these studies, cancers with R1 margins are more likely to have other adverse pathological features, such as extramural venous invasion compared with cancers with R0 margins.^12^ Patterns of relapse also seem to differ according to margin status, with patients with either R1 subtype likely to present with a greater metastatic burden, either in terms of the number of lesions or the number of anatomical sites affected.^13^ Furthermore, the quality of surgical specimens does not appear to differ between cancers with R0 and R1 margins, implying that technical factors may not be a major determinant of margin status.^11^ The fact that the most common form of relapse in patients with either R1tumor or R1LNM margins was distant recurrence in the absence of local recurrence adds further weight to this argument.^13^ Finally, adjuvant therapy appears to be less effective in patients with R1 margins, with the increased risks of relapse persisting even after the use of long course chemotherapy.^14^

Whilst these studies suggest that R1 margins may be a surrogate for more aggressive disease, whether detectable biological differences exist between cancers with R0 and R1 margins remains to be seen. Routine immunohistochemical/molecular analyses, such as those for mismatch repair (MMR) status, offer an opportunity to investigate these potential biological differences.^15^ Mismatch repair status has a recognized stage-adjusted association with prognosis in patients with colorectal cancer, whereby patients with cancers that have deficient MMR (dMMR) pathways have better survival outcomes.^16^ Mismatch repair status also has a predictive value in determining the benefit of adjuvant chemotherapy, with little benefit seen in patients with dMMR cancers compared with surgery alone.^17,18^ However, recent trials of immunotherapy have increased the value of MMR status as a biomarker, as dMMR cancers have been shown to be exquisitely sensitive to immune checkpoint blockade.^19–21^ Therefore, if an association between R1 margins and dMMR status exists, this would not only add further weight to the argument that margin status reflects cancer biology but also be actionable, because these patients could be treated with immunotherapy.

In this study, we sought to investigate the association between MMR status and R1 margins in patients with Stage III colorectal cancers and to determine the impact of MMR status on survival in these patients.

## Methods

This was a retrospective national cohort study and is reported according to STROBE guidelines.^22^ Patients diagnosed with Union for International Cancer Control (UICC) Stage III colorectal cancer in Denmark between January 1, 2016 and December 12, 2019 were identified from the Danish Colorectal Cancer Group (DCCG) database. This national cancer registry includes at least 95% of all patients diagnosed with colorectal cancer in Denmark and has recently been validated, with an overall data accuracy > 95%.^23^

Patients undergoing a potentially curative oncological resection for a single colorectal cancer were included in the study cohort. Patients with synchronous colorectal cancers and those undergoing palliative operations, stents, or local excisions were excluded. Patients also were excluded if MMR status had not been pathologically assessed. Clinicopathological variables were extracted directly from the DCCG database. Patients were stratified into two groups according to MMR status (deficient [dMMR] or proficient [pMMR]). These groups were further subdivided according to tumor location into right colon (cecum, ascending colon, hepatic flexure, transverse colon), left colon (splenic flexure, descending colon, sigmoid colon) and rectum, and according to margin status (R0, R1tumor, R1LNM).

The original pathology assessments from each centre were used for these stratifications. Mismatch repair status was determined by immunohistochemical analysis of the four mismatch repair proteins, MLH1, PMS2, MSH2, and MSH6. Cancers lacking expression of any of these proteins were classified as dMMR. Margin status was defined according to national guidelines by inking of the nonperitonealized radial margins before sectioning of the specimen. R1 resections were defined as the presence of viable cancer cells ≤ 1 mm from the resection margin. These resections were then further divided into those ≤ 1 mm from the primary tumor (R1tumor) and those ≤ 1 mm from a metastatic lymph node or tumor deposit (R1LNM). R0 resections were defined as the absence of viable cancer cells ≤ 1 mm from the resection margin.

The primary outcome of this study was the proportion of dMMR cancers according to margin status (R0 vs. R1tumor vs. R1LNM). Secondary outcomes included overall survival (OS), defined as the time from surgery to the time of death or point of last follow-up, and the association of both MMR and margin status with OS. This study was approved by the Danish Patient Safety Authority (R-20061154) and the Danish Data Protection Agency (P-2020-902).

Univariable analyses of the differences in clinicopathological variables according to MMR status were performed by using the chi-square test for categorical data and the Mann-Whitney-Wilcoxon test for continuous data. All analyses were two-sided and were considered statistically significant with a *p*-value < 0.05. Overall survival was calculated by using the Kaplan-Meier method and compared by using the log-rank test. Univariable and multivariable Cox regression analyses were performed to identify prognostic factors for OS. The following factors were selected for these analyses *a priori*: age, tumor location, pT stage, pN stage, extramural venous invasion (EMVI), lymphatic invasion, perineural invasion, MMR status, and margin status. The results of the Cox regression analyses are presented as hazard ratios (HR) with 95% confidence interval (CI). All analyses were performed by using SPSS version 25.0 (IBM, Armonk, NY).

## Results

### Cohort Demographics

**Fig. 1:**
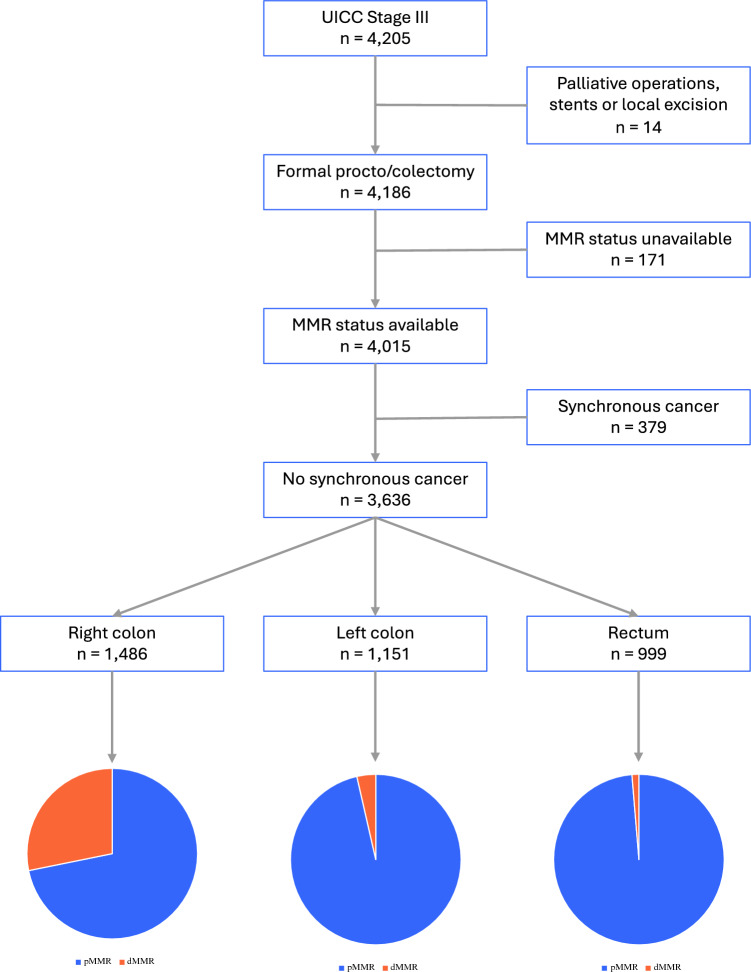
CONSORT diagram of the study cohort. *MMR* mismatch repair status; *dMMR* deficient MMR; *pMMR* proficient MMR

The study cohort comprised at total of 3636 patients with Stage III colorectal cancers (Fig. 1). The demographics of the study cohort are summarized in Table 1. The cohort included 1978 males (54.4%), and the median age was 71 years (interquartile range [IQR] 63–77 years). The median follow-up for the entire cohort was 37 months (IQR 25–51 months). A total of 473 patients (13.0%) had dMMR cancers. Compared with patients with pMMR cancers, these patients were more likely to be older and female. dMMR cancers were significantly more common in the right colon (419, 88.6%) than the left colon (41, 8.7%) and rectum (13, 2.7%, *p* < 0.001). Although an association with more advanced pT stage was seen in patients with dMMR cancers (89.3% ≥ pT3 vs. 81.8% in pMMR cancers, *p* < 0.001), no differences in pN staging (pN1a – 2b) were noted. dMMR status also was more commonly seen in poorly differentiated and mucinous adenocarcinomas and in cancers with lymphatic invasion. There was no evidence that the types of surgical procedures differed between patients with dMMR and pMMR cancers. Emergency operations were performed in 256 patients (7.0%), with no difference in the rate of emergency operations noted according to MMR status (dMMR 6.8% [32 patients] vs. pMMR 7.1% [224 patients], *p* = 0.802). Similarly, no difference in the rate of compromised resections, whereby a less extensive resection was performed due to patient comorbidities, was noted according to MMR status (dMMR 1.7% [8 patients] vs. pMMR 1.2% [37 patients], *p* = 0.339).

**Table 1:** Clinicopathological demographics according to margin status in 3636 patients with Stage III colorectal cancer

		MMR status		
		pMMR	dMMR	
No. patients		3163	473	–
Median age (IQR)		70 (62-77)	74 (68-81)	**< 0.001**
Male:female		1.4	0.5	**< 0.001**
Tumor location	Right colon	1067 (33.7)	419 (88.6)	**< 0.001**
	Left colon	1110 (35.1)	41 (8.7)	
	Rectum	986 (31.2)	13 (2.7)	
Margin status	R0	2682 (84.8)	376 (79.5)	**< 0.001**
	R1tumour	148 (4.7)	42 (8.9)	
	R1LNM	333 (10.5)	55 (11.6)	
pT stage	1	180 (5.6)	13 (2.7)	**< 0.001**
	2	400 (12.6)	38 (8.0)	
	3	1841 (58.2)	285 (60.3)	
	4	742 (23.4)	137 (29.0)	
pN stage	1a	1039 (32.8)	172 (36.4)	0.464
	1b	952 (30.1)	127 (26.8)	
	1c	165 (5.2)	21 (4.4)	
	2a	580 (18.3)	90 (19.0)	
	2b	427 (13.5)	63 (13.3)	
Histological subtype	ADC	2662 (84.2)	178 (37.6)	**< 0.001**
	Poor diff ADC	144 (4.6)	137 (29.0)	
	Mucinous	245 (7.8)	110 (23.3)	
	Signet cell	28 (0.9)	18 (3.8)	
	Other	84 (2.7)	30 (6.3)	
Extramural venous invasion		1435 (45.4)	161 (34.0)	**< 0.001**
Lymphatic invasion		750 (23.7)	149 (31.5)	**< 0.001**
Perineural invasion		863 (27.3)	84 (17.8)	**< 0.001**

Bold values indicate statistically significant

Numbers in parentheses are percentages unless otherwise specified

*MMR* Mismatch repair; *pMMR* Proficient mismatch repair; *dMMR* Deficient mismatch repair; *IQR* Interquartile range; *R0* Microscopically negative margins; *R1tumor* microscopically positive margins to the primary tumor; *R1LNM* microscopically positive margins to lymph node metastases; *ADC* adenocarcinoma

### Association Between Margin Status and MMR Status

When considering the whole cohort, R1 resection margins were found in 15.9% of patients, with R1tumor margins found in 5.2% and R1LNM margins found in 10.7%. The rate of R1 margins was significantly higher in patients with dMMR cancers compared with those with pMMR cancers (20.5% vs. 15.2%, *p* < 0.001), with the greatest difference seen in the rate of R1tumor margins (8.9% vs. 4.7%). Given that both margin status and MMR status varied according to tumor location, further subgroup analyses according to tumor location were performed (Table 2). These demonstrated significantly higher rates of R1tumor margins in dMMR cancers in both right- (8.1% vs. 4.5%) and left-sided colon cancers (19.5% vs. 3.0%) compared with pMMR cancers. No association was seen in patients with rectum cancers, although only 13 of these patients had dMMR cancers.

**Table 2:** Proportion of patients with dMMR cancers according to margin status and tumor location

		MMR		*p*
		pMMR	dMMR	
Right colon	R0	865 (81.1)	335 (80.0)	**0.014**
	R1tumor	48 (4.5)	34 (8.1)	
	R1LNM	154 (14.4)	50 (11.9)	
Left colon	R0	1005 (90.5)	30 (73.1)	**< 0.001**
	R1tumor	33 (3.0)	8 (19.5)	
	R1LNM	72 (6.5)	3 (7.3)	
Rectum	R0	812 (81.3)	11 (1.1)	0.568
	R1tumor	67 (6.7)	0 (0)	
	R1LNM	107 (10.7)	2 (0.2)	

Bold values indicate statistically significant

Numbers in parentheses are percentages unless otherwise specified

*MMR* Mismatch repair; *pMMR* Proficient mismatch repair; *dMMR* Deficient mismatch repair; *R0* Microscopically negative margins; *R1tumor* microscopically positive margins to the primary tumor; *R1LNM* microscopically positive margins to lymph node metastases

To investigate whether the rates of R1 resection margins differed between patients with sporadic or Lynch-associated dMMR cancers, subgroup analyses of patients with dMMR cancers were performed. The group was divided according to age (< 75 years [239 patients] vs. ≥ 75 years [234 patients]), because sporadic dMMR cancers are more common in elderly patients.^24^ No difference in the rate of R1 resection margins were noted in these subgroup analyses (< 75 years 19.7% vs. ≥ 75 years 21.4%, *p* = 0.647), with similar rates of R1tumor (7.9% vs. 9.8%) and R1LNM margins (11.7% vs. 11.5%) in each group.

### Association Between MMR Status and Survival

**Fig. 2:**
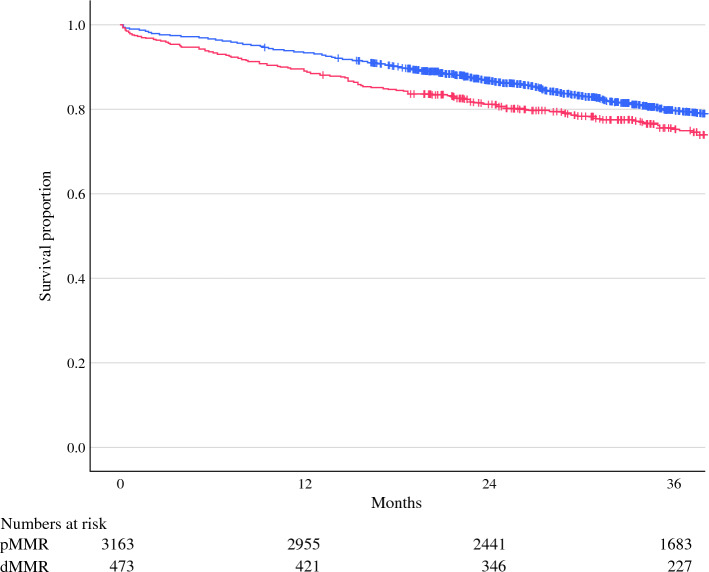
Overall survival (OS) stratified according to mismatch repair (MMR) status. The blue line represents patients with pMMR cancers (3-year OS 79.7% [95% CI 78.9–80.5]). The red line represents patients with dMMR cancers (3-year OS 75.3% [95% CI 73.2–77.4], *p* = 0.023).

**Fig. 3:**
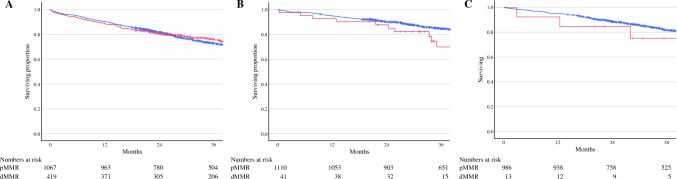
Overall survival (OS) stratified according to mismatch repair (MMR) status and tumor location. **A** Right-sided colon cancers—3-year OS in patients with pMMR cancers (blue line) 72.9% (95% CI 71.5–74.3) versus dMMR cancers (red line) 75.8% (95% CI 73.6–78.0), *p* = 0.246. **B** Left-sided colon cancers—3-year OS in patients with pMMR cancers (blue line) 84.5% (95% CI 83.4–85.6) versus dMMR cancers (red line) 69.9% (95% CI 61.5–78.3), *p* = 0.029. **C** Rectum cancers—3-year OS in patients with pMMR cancers (blue line) 81.7% (95% CI 80.4–83.0) versus dMMR cancers (red line) 75.2% (95% CI 62.6–87.6), *p* = 0.598

**Fig. 4:**
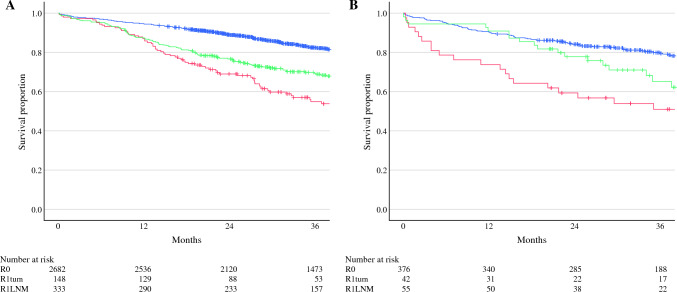
**A** Overall survival (OS) in patients with pMMR cancers. Three-year OS in those with R0 margins (blue line, 82.4% [95% CI 81.6–83.2]), R1tumor margins (green line, 56.0% [95% CI 51.6–60.4]) and R1LNM margins (red line, 69.3% [95% CI 66.7–71.9]). **B** Overall survival (OS) in patients with dMMR cancers. Three-year OS in those with R0 margins (blue line, 79.5% [95% CI 77.3–81.7]), R1tumor margins (green line, 51.0% [95% CI 43.1–58.9]) and R1LNM margins (red line, 65.2% [95% CI 58.1–72.3])

When considering the whole cohort, patients with pMMR cancers had significantly better overall survival (OS) compared with those with dMMR cancers (3-year OS 79.7% [95% CI 78.9–80.5] vs. 75.3% [95% CI 73.2–77.4], *p* = 0.023) (Fig. 2). However, when stratified according to tumor location, the association with pMMR status and improved OS was only seen in patients with left-sided colon cancers (3-year OS 84.5% [95% CI 83.4–85.6] vs. 69.9% [95% CI 61.5–78.3], *p* = 0.029) and not in those with right-sided colon cancers (3-year OS 72.9% [95% CI 71.5–74.3] vs. 75.8% [95% CI 73.6–78.0], *p* = 0.246) (Fig. 3). The effect of margin status on survival outcomes in patients with pMMR (Fig. 4a) and dMMR (Fig. 4b) cancers also was investigated. In both groups, R1 margins were associated with significant reductions in 3-year OS compared with R0 margins, with the worst outcomes seen in patients with R1tumor margins. In patients with R1tumor margins, no difference was noted in survival between patients with dMMR or pMMR cancers (3-year OS 51.0% [95% CI 43.1–58.9] vs. 54.9% [95% CI 50.5–59.3], *p* = 0.388). Following multivariable analysis, no significant association between MMR status and OS was identified (Table 3). Age, pN stage, EMVI, perineural invasion, and R1 margins were associated with poorer OS, whereas left-sided cancers were associated with improved OS.

**Table 3:** Univariable and multivariable analyses of prognostic factors for overall survival

		HR (95% CI)	*p*	HR (95% CI)	*p*
Age		**1.07 (1.06-1.08)**	**<0.001**	**1.07 (1.06-1.08)**	**<0.001**
Tumor location	Right	Reference	–	Reference	–
	Left	**0.57 (0.48-0.67)**	**<0.001**	**0.70 (0.57-0.87)**	**0.001**
	Rectum	**0.65 (0.55-0.77)**	**<0.001**	0.94 (0.76-1.16)	0.581
T stage	1	Reference	–	Reference	–
	2	1.40 (0.83-2.35)	0.202	0.85 (0.47-1.53)	0.590
	3	**2.21 (1.39-3.49)**	**<0.001**	0.86 (0.52-1.45)	0.577
	4	**4.68 (2.95-7.44)**	**<0.001**	1.39 (0.81-2.37)	0.230
N stage	1a	Reference	–	Reference	–
	1b	1.14 (0.94-1.38)	0.174	1.00 (0.79-1.26)	0.983
	1c	1.30 (0.91-1.85)	0.145	1.01 (0.70-1.45)	0.976
	2a	**1.61 (1.32-1.96)**	**<0.001**	**1.38 (1.08-1.77)**	**0.011**
	2b	**2.70 (2.22-3.28)**	**<0.001**	**1.77 (1.37-2.29)**	**<0.001**
EMVI		**2.05 (1.79-2.36)**	**<0.001**	**1.38 (1.15-1.66)**	**<0.001**
Lymphatic invasion		**1.58 (1.34-1.86)**	**<0.001**	*	*
Perineural invasion		**1.87 (1.62-2.16)**	**<0.001**	**1.43 (1.19-1.72)**	**<0.001**
MMR	pMMR	Reference	–	Reference	–
	dMMR	**1.24 (1.03-1.51)**	**0.023**	*	*
Margins	R0	Reference	–	Reference	–
	R1tumour	**2.86 (2.29-3.59)**	**<0.001**	**2.16 (1.65-2.83)**	**<0.001**
	R1LNM	**1.97 (1.64-2.38)**	**<0.001**	**1.47 (1.16-1.86)**	**0.001**

Bold values indicate statistically significant

*EMVI* Extramural venous invasion; *MMR* Mismatch repair; *pMMR* Proficient mismatch repair; *dMMR* Deficient mismatch repair; *R0* Microscopically negative margins; *R1tumor* Microscopically positive margins to the primary tumor; *R1LNM* Microscopically positive margins to lymph node metastases

## Discussion

To the authors’ knowledge, this is the first study to investigate the association between MMR status and R1 resection margins in patients with Stage III colorectal cancers. We found that patients with dMMR cancers have significantly higher rates of R1tumor margins following potentially curative surgery and that this association is seen in both patients with right- or left-sided colon cancers. These findings should have a substantial clinical impact. The association with R1 margins and poorer oncological outcomes has long been recognized in patients with rectum cancers.^2–5^ However, recent studies have highlighted that R1 margins occur just as frequently in patients with colon cancer.^6–8^ These studies demonstrate a consistent association between R1 margins and increased risks of systemic relapse and death in these patients. Recent studies by our group, using the same patient cohort as in the current study, demonstrated that these risks are greater in patients with R1tumor margins compared with those with R1LNM margins.^12,13^ As such, patients with R1 resections after potentially curative surgery for colon cancers should be considered a particularly high-risk group.

One possible way to address these risks would be to escalate therapy in these high-risk patients. This should ideally be in the form of neoadjuvant therapy; in a previous study, we found that the increased risks of relapse in patients with R1 resections persist even after long-course adjuvant chemotherapy.^14^ Although neoadjuvant chemotherapy does not yet have an established role in the treatment of colon cancers, the FOxTROT study has provided some promising results.^25^ In that study, patients treated with neoadjuvant oxaliplatin-fluoropyrimidine chemotherapy had a significantly lower rate of R1 resections following surgery compared with the control arm (6% vs. 11%, *p* < 0.001). However, FOxTROT also highlighted two potential issues with neoadjuvant chemotherapy. The first lies in patient selection. Clinical staging for colon cancer has limited accuracy.^26^ By using clinical staging to allocate “high-risk” patients to neoadjuvant chemotherapy, FOxTROT was associated with significant risks of overtreatment, whereby 24% of patients in the control arm were found to have Stage I or low-risk Stage II cancers. Similar risks of overtreatment were seen in the PRODIGE 22 study.^27^ So, whilst neoadjuvant chemotherapy may be of some benefit in patients with colon cancer, identifying which patients are at high-risk before surgery is challenging. This is even more true for patients with R1 margins, a characteristic that by definition is only known after surgery.

The second issue with neoadjuvant chemotherapy is that it appears only to be of benefit in patients with pMMR rather than dMMR cancers.^25^ Fortunately, contrasting results have been shown in early trials of neoadjuvant immunotherapy. In the NICHE study, a single dose of ipilimumab and two doses of pembrolizumab resulted in major pathological responses in 95% of patients with dMMR cancers compared with 20% of patients with pMMR cancers.^20^ Similarly impressive results have been seen in patients with dMMR rectal cancers, where in an early clinical trial of 9 cycles of treatment with dostarlimab resulted in clinical complete responses in all patients, obviating the need for further treatment.^19^ Although these results need to be confirmed in larger trials, the exquisite sensitivity of dMMR colorectal cancer to immune checkpoint blockade has already led many to question whether surgery is still necessary in the majority of these patients. Whilst this remains to be seen, what is undoubtable is that neoadjuvant immunotherapy offers a clear opportunity to avoid R1 resections in patients with dMMR cancers, who can easily be identified by preoperative immunohistochemical/molecular analyses of diagnostic biopsies. Based on the proportion of patients with dMMR cancers in the current study, such a strategy would reduce the total number of R1 resections in patients with Stage III colorectal cancer by > 15%.

Despite its association with R1 resections, dMMR status was not found to be independently associated with survival in the current study. While this may seem paradoxical, there have been conflicting reports about the prognostic value of dMMR in previous studies. In general, dMMR cancers have an association with an earlier stage of disease at the time of diagnosis compared with pMMR cancers.^28^ Furthermore, in patients with Stage II disease, there is robust evidence that dMMR cancers are associated with reduced risks of relapse and improved survival.^29^ However, in patients with Stage III disease, two meta-analyses have found contrasting results and any association with survival outcomes is less certain.^30,31^ Finally, in patients with metastatic disease, dMMR status actually appears to be associated with poorer oncological outcomes.^32^ The reason for these contrasting associations with survival is not entirely clear. Given the association of dMMR status with increased immune infiltration of the primary tumour, one hypothesis is that stage progression is associated with exhaustion of any antitumor immune responses, annulling the prognostic advantages seen in earlier-stage disease.^33,34^

The authors recognise the limitations of this study. In Denmark, MMR status is determined by using immunohistochemistry. Other molecular assessments, such as for BRAF mutations, are not mandatory during the initial diagnostic workup. As such, it was not possible to divide the patients in the current study into those with sporadic dMMR cancers and those with hereditary dMMR cancers associated with Lynch syndrome. Significant differences have been demonstrated between these groups, whereby sporadic dMMR cancers are more common, more likely to occur in elderly patients and are associated with poorer survival.^24^ As such it would have been of interest to investigate whether the association with R1 margins is seen in both dMMR subgroups. The subgroup analyses performed in the current study were based on an arbitrary age cutoff of 75 years, as the majority of patients diagnosed with dMMR cancers over this age have sporadic cancers. While these analyses did not find any differences in the rate of R1 margins between these groups, these results should be interpreted with caution. A more robust approach would have been to compare outcomes between sporadic and hereditary dMMR cancers defined by molecular assessments. A further limitation is that whilst survival data was available for these patients, corresponding data regarding both local and distant recurrence was not available. Although one may argue that survival is the most relevant endpoint in these patients, it would have been of interest to investigate the impact of MMR status on disease recurrence as well.

## Conclusions

In patients with Stage III colon cancers, dMMR status is associated with a significantly higher risk of R1tumor margins following potentially curative surgery. This adds a further rationale for the use of neoadjuvant immunotherapy in this patient group.
